# Structure and Composition of a Novel Refractory Ni-Containing CrMoNbTaVW High-Entropy-Alloy Thin Film

**DOI:** 10.3390/ma19040675

**Published:** 2026-02-10

**Authors:** Dimitri Litvinov, Jarir Aktaa, Adam Bichler, Michael Stueber, Sven Ulrich

**Affiliations:** Institute for Applied Materials (IAM), Karlsruhe Institute of Technology (KIT), Hermann-von-Helmholtz-Platz 1, 76344 Eggenstein-Leopoldshafen, Germany; jarir.aktaa@kit.edu (J.A.); adam.bichler@tuwien.ac.at (A.B.); michael.stueber@kit.edu (M.S.); sven.ulrich@kit.edu (S.U.)

**Keywords:** refractory high-entropy alloy, thin films, magnetron sputtering, electron microscopy

## Abstract

The structure and composition of a refractory Ni-containing CrMoNbTaVW high-entropy-alloy (HEA) thin film were investigated. The HEA thin film with a thickness of 5 μm was grown via conventional direct current magnetron sputtering from a multiple-elemental compound target. The Ni-containing HEA thin film with a Ni concentration of 3.6 at. % exhibits a single-phase body-centered cubic (BCC) crystal structure with a lattice parameter of *a* = 0.316 nm. The grains in the HEA thin film are columns, extended in the growth direction. They are not aligned exactly perpendicular to the substrate surface. The thin film grows in a polycrystalline structure with a tendency to preferred orientation or texture. Energy-dispersive X-ray analyses of the HEA thin film show near-equal atomic concentrations of Cr, Mo, Nb, Ta, V, and W elements in the range 15–17 at. % with almost uniform distribution. In contrast, Ni is not uniformly distributed in the film, and grains with a different Ni concentrations were observed. The defects observed in the HEA thin film are mainly single dislocations or an assembly of dislocations, which could be caused by residual stresses in the layer forming during the growth of the HEA thin film.

## 1. Introduction

High-entropy alloys (HEAs) consisting predominantly of refractory elements with high melting points such as Hf, Mo, Nb, Ta, V, Zr and W, are generally considered to be refractory HEA and are discussed in the literature with strong emphasis. Refractory HEAs, first proposed by Senkov et al. in 2010 [1], have attracted attention due to their versatile properties, such as good mechanical properties, excellent anti-oxidation performance, outstanding corrosion resistance, and high thermal stability, indicating significant application potential, both in form of bulk and thin-film materials [1,2,3,4,5,6,7,8,9,10]. Individual multiple-principal-element-alloy bulk and thin-film materials representing different mixtures of four, five or six refractory metals of the group Cr, Mo, Nb, Ta, V and W have already been thoroughly and systematically investigated [1,2,3,4,5,6,7]. For example, MoNbTaW multiple-principal-element alloys and MoNbTaVW HEA with equiatomic concentrations of elements prepared via arc-melting have been shown to form a single-phase body-centered cubic (BCC) structure and to maintain chemical homogeneity with good mechanical properties [1,2,3]. Different 5-elemental refractory HEA thin films based on MoNbTaW + X systems with thicknesses of 300–400 nm and grown using magnetron sputtering were investigated in [4]. Here, the additional element X used was Ti, V, Cr, Mn or Hf, and all films showed a BCC structure with a lattice parameter in the range of 0.32–0.33 nm and a dense columnar morphology. In [5], it was shown that the refractory HEA thin films based on the multi metal system MoNbTaW are promising materials for high-temperature applications where high thermal stability is important. Moreover, 6-elemental CrMoNbTaVW HEA thin films with a single-phase BCC structure, created using combinatorial synthesis, were reported in [6] along with a study of the mechanical properties of the thin films in dependence on different concentrations of elements. In [7], the authors predicted on the basis of thermodynamical calculations and simulations that Cr_x_MoNbTaVW HEA can be tailored over a certain range of Cr content with the BCC structure.

The pronounced high room-temperature brittleness of refractory HEAs is, however, a key factor restricting their application. Previous studies have shown that the addition of Ti and Ni can effectively reduce the room temperature brittleness of refractory HEAs. Zhang et al. [11] investigated the effects of adding Ti and Ni on the microstructure and high-temperature mechanical properties of NbMoTa-based refractory HEAs and showed improvement of the mechanical properties of the alloy upon this chemical modification. This emerging field of new, specifically alloyed HEA materials has not yet been systematically explored.

In this study, the microstructural characterization of a magnetron-sputtered 6-elemental CrMoNbTaVW thin film of near-equiatomic concentration and modified with a small amount of Ni was carried out via X-ray diffraction, scanning and transmission electron microscopy. When developing HEA, one approach is to combine only elements exhibiting the same crystal structure, which limits the number of elements that can be used. This work uses the refractory metals of the fifth subgroup, V, Nb, and Ta, as well as of the sixth subgroup, Cr, Mo, and W, all of which have a BCC crystal structure. The bonds are predominantly metallic, but also covalent, which could have a positive effect on the hardness and strength of the forming alloy. If the entropy is to be increased further, additional elements with the same crystal structure must be added. In the PSE, only the elements Fe, Eu, and Fr, as well as the alkali metals Li, Na, K, Rb, Ce, and the alkaline earth metals Ca, Sr, and Ba, have a body-centered cubic crystal structure. The alkali and alkaline earth metals mentioned above are excluded because their high ionic bonding content may have a negative effect on mechanical properties. Eu from the lanthanide group is excluded as a rare earth metal due to its high price of approximately EUR 3000 per kg, its high chemical reactivity, low melting point of 1099 K (826 °C) and poor mechanical properties. The alkali metal Fr is excluded because it is radioactive. Fe is less suitable because it is not resistant to corrosion and oxidation. This raises the question of how additional elements can be added. One possible way is to alloy an element that does not have a BCC crystal structure but is soluble in a BCC crystal structure. As the number of HEA elements used increases, in this case, Cr, Mo, Nb, Ta, V, and W, the average concentration of the individual elements decreases with uniform distribution, in this case 16.7 at. %; it thus approaches the still unknown solubility limit of this HEA. It should also be noted that the physical vapor deposition (PVD) process utilized in this study can significantly extend the solubility limit because the synthesis of thin-film materials can take place far from thermodynamic equilibrium. For example, the maximum solubility of Al in cubic face-centered TiN is approximately 5 at. %. Using a PVD process, the maximum solubility could be increased to 76 at. % for a metastable, cubic face-centered, single-phase (Ti,Al)N solid solution-type crystal. Such (Ti,Al)N thin film materials are widely used, for example, in manufacturing and tooling applications; they exhibit high thermal and thermochemical stability. This highlights the major difference between thin-film synthesis and methods used to produce bulk alloys. The cooling rate in PVD synthesis is significantly higher, at up to 1010 K/s, and such metastable materials can only be produced up to their maximum solubility.

This study is the initial result of a more recent, significantly more comprehensive research study that will investigate the influence of Ni concentration on the formation of the microstructure of Ni-containing high-melting alloys in the CrMoNbTaVW system over a wider range. We investigate whether Ni can be completely dissolved under defined synthesis conditions in a single-phase cubic face-centered six-element refractory alloy with a concentration of 3.6 at. %, whether a second fcc Ni phase forms, or whether Ni enrichment or depletion occurs at the grain boundaries. The aim is to use the kinetics of physical vapor deposition so that Ni does not crystallize in the face-centered cubic structure that forms under thermodynamic equilibrium conditions but instead dissolves in the bcc lattice. This synthesis concept could prove to be an interesting tool for the development of metastable single-phase HEA materials.

## 2. Material and Experimental Techniques

The investigated thin film with a thickness of ~5 μm was grown via d.c. magnetron sputtering from a multiple-elemental compound target with concentrations of Cr, Mo, Nb, Ta, V, W in the range 11–20 at. % and ~10 at. % of Ni. The target diameter was 75 mm, and the target power was 500 W d.c. The thin film deposition was carried out on cemented carbide substrates (WC-Co with 11 wt.% of Co) in a pure argon atmosphere with an argon gas flow of 30 sccm and a pressure of 0.2 Pa. The substrate temperature was 350 °C. The substrates were grounded during deposition. The residual gas pressure was 0.32 mPa. Before the actual coating process started, the substrates were ultrasonically cleaned in acetone and plasma-etched with argon ions.

Structural characterization of thin films was carried out via X-ray diffraction (XRD), scanning (SEM) and transmission electron microscopy (TEM). TEM investigations were carried out using a Talos F200X microscope (Thermo Fisher Scientific Inc., Waltham, MA, USA) operating at 200 kV equipped with a high-angle annular dark-field (HAADF) detector for scanning TEM (STEM) mode. TEM cross-section samples were prepared using a focused ion beam method. For the determination of the thin film composition, energy-dispersive X-ray (EDX) spectroscopy was used. For the crystallographic investigations, XRD on a Seifert X-ray diffractometer Space Universal (XRD Eigenmann GmbH, Schnaittach, Germany) with Cu-Kα radiation, conventional TEM with selected area electron diffraction, and high-resolution TEM imaging (HRTEM) with fast Fourier transformation (diffractogram) were applied. The experimental parameters for XRD analysis were a 2Theta range of 30° to 135° with step size of 0.02°. SEM investigations were carried out using a Zeiss EVO MA 10 microscope (Carl Zeiss IQS Deutschland GmbH, München, Germany) equipped with a Bruker EDX detector at 20 kV (Bruker Nano GmbH, Berlin, Germany). The standard deviation of determination of atomic concentrations with EDX only from thin film for major components was not more than ±5% relative.

Indentation with a Vickers indenter was performed using an Anton Paar micro-combi tester force-controlled system (Anton Paar Germany GmbH, Ostfildern, Germany) with a maximum test force of F = 500 mN. The loading rate was F = 1000 mN/min, and the unloading rate was F = 2000 mN/min. The maximum force was held for t = 30 s. The corresponding force-displacement curves were recorded. A total of nine impressions were produced on the sample, and the mean values of the parameter were calculated from the individual measurements. The reduced modulus of elasticity (Er) was determined by means of indentation. This accounts for the fact that elastic deformations occur in the sample and in the test specimen. Based on the Stoney equation, it is possible to infer the mechanical stress of the thin film from the substrate curvature resulting from a coating process. To do this, the curvature of the uncoated and coated substrate must be measured [12].

## 3. Results and Discussion

### 3.1. Surface Morphology of the Ni-Containing CrMoNbTaVW Thin Film

**Figure 1:**
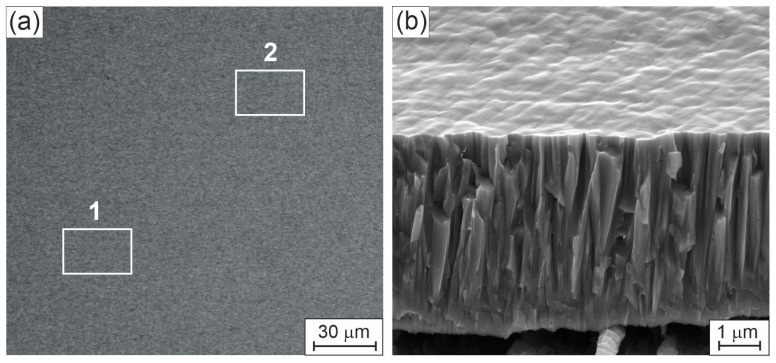
SEM images of the HEA thin film: top-view image of the surface on large scale (**a**), and fracture cross-section and surface view on small scale (**b**). Areas marked by white rectangles in (**a**), specified as areas 1 and 2, reflect areas of EDX measurements, presented in Table 1.

Figure 1 shows SEM images of the surface (Figure 1a) and fracture surface (Figure 1b) of the investigated HEA thin film on different scales.

The top-view image on large scale in Figure 1a shows a very smooth and homogeneous surface of the HEA thin film. This is also confirmed by the SEM image of the fracture cross-section and surface on a smaller scale in Figure 1b: it is clear that the thin film exhibits a surface topography with significant growth features, i.e., a homogeneous, dense columnar structure is indicated. No coarse impurities, growth defects, or pores are visible. The cross-sectional thin film morphology is smoother in substrate-near areas compared to areas further away from the substrate and closer to the film surface. This reflects a characteristic V-shaped columnar growth, as is common for conventional magnetron sputtered thin films. The columnar structure of the HEA thin film is supported by further TEM observations (see below, Figure 3 and Figure 4c), which clearly show that the grains in the HEA thin film are columnar with diameters up to few hundred nm. The elemental SEM-EDX maps reveal a homogeneous distribution of all elements on both large and small scales. Note that the SEM-EDX signal is obtained from a large area of the layer with a depth up to 1 μm, which leads to a bulk-like average in the determination of the elemental concentrations. Table 1 presents the results of the EDX measurements on the surface in two different areas 1 and 2 of the HEA thin film in Figure 1a and indicated by white rectangles with sizes of ca. 30 μm × 20 μm.

We observe almost equal atomic concentrations of the six metallic elements Cr, Mo, Nb, Ta, V and W in the HEA thin film in the range 15–17 at. % and a small amount of Ni, ~3.6 at. %. It is obvious that the elemental composition of the thin film deviates significantly from that of the sputter target. In particular, the Ni concentration of the film is much lower in the HEA thin film than in the target. An explanation for this can be found in the plasma conditions applied for the magnetron sputter deposition, which clearly depends on gas pressure, gas flow, substrate temperature, target-to-substrate distance and magnetic field configuration at the target. A deviation in the elemental composition of a deposited thin film and the related target has frequently been observed in non-reactive magnetron-sputtered thin films, especially when the material to be deposited consists of light-weight and heavy-weight atoms. There are several effects that overlap in complex ways. First, the sputtered particles on the target have different angles and energy distributions; second, the cross sections for the collision processes in the plasma are also different; and third, the sticking coefficients of the incoming particles on the substrate surface are also different. All processes depend on the energy and the collision parameter. For example, it has been a long-standing challenge to deposit stoichiometric transition metal diboride thin films from perfect stoichiometric diboride compound targets. In the case of titanium diboride, TiB_2_, numerous publications in recent decades referred to either sub- or over-stoichiometric thin films, TiB_2−x_ or TiB_2+y_ (with x, y in the range of 0.1 up to 0.7 or similar). Neidhardt et al. [13], Petrov et al. [14], and Bakhit et al. [15] offered the key to understanding the behavior and flow of sputtered atoms with high and low atomic mass in relation to plasma conditions, pressure, mean free path of species in a gas, interaction with electric and magnetic fields and more; they demonstrated how to tune the deposition process to tailor thin-film composition and to deposit perfectly stoichiometric diboride thin films [13,14,15]. Similarly, the composition of ruthenium aluminide thin films with B2 structure can be easily modified within the existence range of this phase by magnetron sputtering of a stoichiometric RuAl 50/50 target (with very high atomic mass (Ru) and low atomic mass (Al)) by simple variation of the gas pressure in correlation to the target-to-substrate distance and other plasma parameters [16]. Rausch et al. [17] investigated Ni-W thin films produced from a Ni/W target using d.c. magnetron sputtering and found, among other things, that the Ni content in these coatings was also lower than the Ni content of the target, which agrees with our measurements. In order for the coatings to have almost the same composition as that of a multi-component target, it is necessary to work at a very high pressure between 1 Pa and 10 Pa so that many collisions take place in the plasma. However, this comes at the cost of the formation of many nanovoids, which usually leads to tensile residual stresses. However, in this publication, a low Ni content is targeted in order to find out whether a single-phase microstructure still forms. For this reason, a low working gas pressure of 0.2 Pa was specifically chosen. In the case of the complex Ni-containing alloy presented here, we identified conditions for the deposition, where the heavy elements Mo, W, Ta, and Nb behave different form the lighter ones, Cr, Ni, and V. Under a well-defined combination of pressure, temperature, target operation conditions and target-to-substrate distance, we observed the formation of thin films with the reported single-phase solid-solution structure, incorporating 3.6 at. % Ni.

### 3.2. Structure of the Ni-Containing CrMoNbTaVW Thin Film

**Figure 2:**
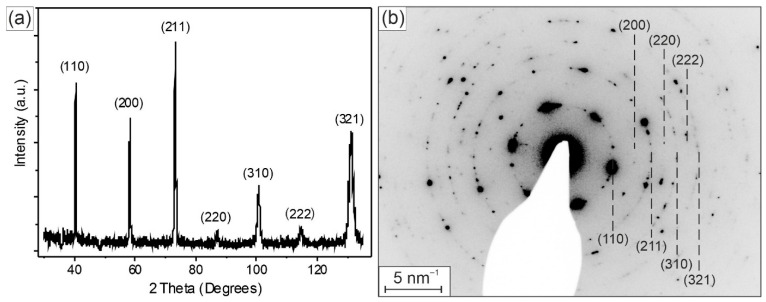
(**a**) X-ray and (**b**) electron diffraction patterns of the Ni-containing HEA thin film. The indexed peaks belong to a BCC crystal lattice with lattice parameter *a* = 0.316 nm.

Figure 2 shows the X-ray diffraction (a) and electron diffraction (b) patterns of the HEA thin film.

All diffraction peaks on the X-ray diffraction pattern obtained from the Ni-containing HEA thin film in Figure 2a and the diffraction circles and spots from the large area of the thin film cross-section TEM sample in Figure 2b have been identified as belonging to a perfect body-centered cubic (BCC) phase. The indexes of the crystal planes corresponding to the XRD peaks are indicated in Figure 2a. Usually, for uniform homogeneous materials, the intensity of the XRD peaks decreases with increasing lattice plane indexes. Texture can be recognized as an enhancement of the relative intensity of some Bragg reflections and a reduction of others. In Figure 2a, we observe a higher intensity of the (110), (211) and (321) peaks, which reveals a tendency of the HEA thin film to a growth with the preferred orientation or even the presence of textures of these crystal planes. The identical lattice planes of the BCC crystal structure are displayed and indexed in the electron diffraction pattern in Figure 2b, obtained from large area of the cross-section TEM sample. Here, in addition to strong (110) spots, we also observe many spots of (211) and (321) reflections, which also supports the statement about its texture. The lattice parameter of the BCC phase, determined using Figure 2a,b, is *a* = 0.316 ± 0.001 nm.

**Figure 3:**
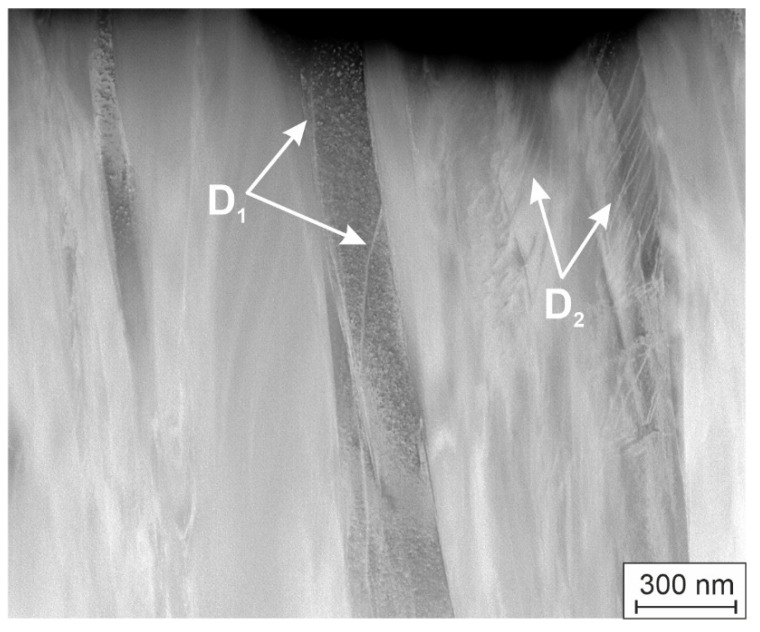
HAADF STEM image of the HEA thin film. Dislocations are marked by D_1_ and D_2_.

Figure 3 shows a cross-section HAADF image of the HEA thin film, which was oriented parallel to the substrate surface (horizontal edge).

The grains of the thin film are columns, extended in the growth direction but not aligned exactly perpendicular to the substrate surface. The width of the grains is changed in the growth direction and reaches a few hundred nm. This geometrical appearance of the V-shaped columnar grains matches the growth of magnetron sputtered thin films. Furthermore, we observe dislocations inside the grains. These are single dislocations inside the grains (see a grain in Figure 3 on the left side with dislocations indicated by white arrows and marked as D_1_), while other grains contain assemblies of the dislocations (see a grain in Figure 3 on the right side with dislocations indicated by white arrows and marked as D_2_), which are parallel and arranged in the same planes. Similar groups of dislocations in another area of the thin film are shown in Figure 6 (see below). This phenomenon is often observed when compressive residual stresses have developed during the growth of the thin films. Such a dislocation pattern indicates yielding due to residual stresses which have developed during the growth of the thin films and obviously reached the yield strength fulfilling the yield criterion. The thin film stresses are estimated by characterizing the deformation of a coated Si-wafer model substrate and by utilizing the Stoney equation accordingly. The nature of the stress of the Ni-containing thin film is compressive, and the order of magnitude is about −800 MPa, which is in the order of the yield strength of similar materials. If the film-forming particles exhibit only low surface diffusion, nanovoids can form and the residual stresses would tend to be tensile. If thin layers do not exhibit tensile residual stress, it can be assumed that the surface diffusion of the film-forming particles is large enough and no nanovoids exist. In this study, the substrate temperature was 350 °C, and the observed residual stress is compressive, while its level is relatively low (<1 GPa). However, not many details on diffusion in such complex materials are known, and the thin film tends to grow with a preferred orientation. Therefore, it is difficult to draw a clear conclusion at this stage. However, we can state that the substrate was grounded during deposition, i.e., the nucleation and subsequent growth of the thin film was not affected by major argon ion bombardment. Thus, we can assume that the forming residual compressive stress may be influenced by thermal stresses, growth effects (i.e., competing grain growth) and by configurational effects, i.e., by incorporating the small fraction of Ni atoms in the single-phase BCC structure of the thin film.

**Figure 4:**
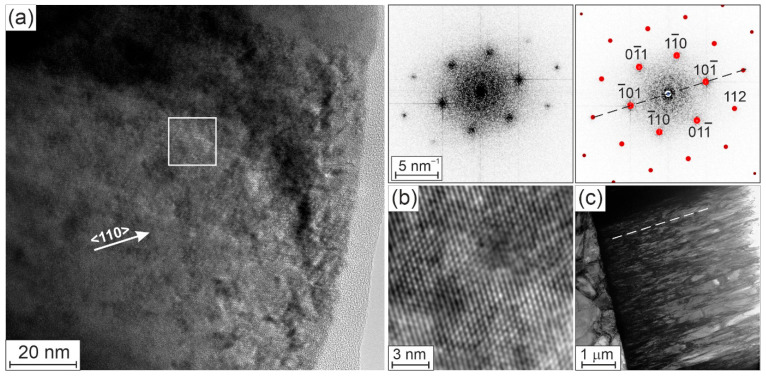
(**a**) HRTEM image of the HEA thin film close to the [111]-zone axis orientation of the BCC structure with measured (**right**) and simulated diffractograms of the area marked by a white square, (**b**) filtered enlarged HRTEM image, (**c**) bright-field overview image with substrate (**left**) and HEA thin film (**right**). The dashed lines show the <110> crystal direction of the HEA thin film.

Figure 4a shows a HRTEM image of the HEA thin film with evaluated (left-hand-side) and simulated (right-hand-side) diffractograms from the area marked by the white square in the HRTEM image and the corresponding enlarged point pattern of the HRTEM image (Figure 4b). The simulation of the diffractogram shows a BCC crystal structure close to the [111]-zone axis orientation.

In Figure 4a, we see only a part of a columnar grain, which is elongated in the growth direction and cropped from the top during the FIB preparation of the TEM sample. The comparison of the HRTEM image in Figure 4a with the identically oriented bright-field overview image in Figure 4c shows that the grain in Figure 4a is elongated in the <110> crystal direction of the HEA thin film.

It is known that, in pure BCC metals the {110} crystal planes have a lowest surface energy [18]. Raabe [19] has observed {110}, {211} and {321} rolling textures of BCC metals in his simulations and related those to the activation of slip systems. In the BCC crystal structure, which was clearly identified as the only crystalline phase of the investigated HEA thin film, there are three slip systems that can be activated during plastic deformation: {110}<111>, {211}<111>, and {321}<111>. The activation of these slip systems depends on factors such as the temperature, strain rate, chemical composition, and crystal orientations [20]. The slip in a BCC structure always occurs in the closest packed ⟨111⟩ direction. As an example, in BCC Ta slip is predominantly on the {110} planes at low temperatures. As temperature increases, slip is observed on the {110}, {211} and {321} planes at room temperature and higher temperatures. The transition temperature from the {110} to the {112} slip has been found to be between 100 K and room temperature, especially for tantalum materials [21,22,23]. The dominant dislocation slips on the {211} planes were experimentally observed in tantalum single crystals under compression at room temperature [22]. The breakdown of so-called Schmid’s law (slip on {110} plans) was reported for group V and VI BCC transition metals, via numerous atomistic simulations and physically informed continuum crystal plasticity model studies [23,24]. The main reason for non-Schmid behavior in transition metals was revealed to be the cores of 1/2<111> screw dislocations spreading on various {110} planes in the <111> zone.

In many component BCC crystals, consisting of group V and VI BCC refractory metals, the slip is not studied in detail. Thus, in the case of 4-component NbMoTaW columnar thin films [25,26], it has been generally observed that slip activity predominantly occurs on {110} planes at room temperature. The columns were mainly extended in the <110> growth direction.

Our investigated complex HEA thin film contains six refractory metals with an additional small Ni amount. It has also a perfect BCC structure. The increased intensities of some XRD peaks in Figure 2a and the presence of solid spots in the electron diffraction pattern in Figure 2b reveal the presence of a texture of {110}, {211} and {321} crystal planes in the investigated HEA thin film. In general, a texture can arise due to templating from the crystalline orientation of the substrate, minimizing the surface energy during deposition, or the competition between surfaces with anisotropic growth rates from grains with different orientations. In our case, the substrate has large grains with sizes larger than 1 μm (Figure 4c). The different crystal planes of the substrate grains can come to the substrate–EA thin film interface. We carried out XRD analyses of the pure substrate and found that one peak from the crystal planes of the substrate exactly corresponds to the {211} peak of the investigated HEA layer. This means that there are crystal planes in the substrate and in the HEA thin films with the same lattice plane distance. Close to the XRD {321} peak of the HEA thin film, there is also a small peak from the substrate. The other peaks are far apart. The preferred alignment of the three crystal orientations {110}, {211} and {321} is caused by minimization of surface energy, competitive columnar growth and strain-energy minimization. Epitaxial effects can be ruled out because the WC-Wo substrate is polycrystalline. Jansson et al. [27] came to a similar conclusion. They investigated magnetron-sputtered CoCrFeMnNi coatings on polycrystalline 316 L steel substrates. They showed that the orientations of the substrate grains have a profound impact on the growth of the coating. This leads to different microstructures and surface morphologies on different substrate grains. Since the sub-states were polycrystalline here too, no epitaxial growth could be observed when looking at the entire substrate.

From the other side, during the growth of the HEA thin film, the columnar grains also extend in the perpendicular direction and come into contact with different crystal planes, which leads to stress or deformation in the layer. The deformation mechanisms in BCC materials are primarily related with slip deformation. In the investigated HEA thin film, we observe assemblies of dislocations in Figure 3 and Figure 6, which are arranged on {110} slip planes. This reveals plastic deformation inside the layer during the growth of the different textured grains. Thus, the activation of all the slip systems may play a role in the formation of {110}, {211} and {321} textures in the investigated HEA thin films. The investigated coatings exhibit compressive internal stress. The thin film attempts to minimize energy by forming a texture, because certain planes are easier to compress. To achieve this, all slip systems are exploited. The activation of all slip systems may play a role in the formation of {110}, {211} and {321} textures, which, in turn, may facilitate the activation of the slip systems. The textures observed in the investigated HEA thin films could thus be a result of this mutual influence.

### 3.3. Composition of the Ni-Containing CrMoNbTaVW Thin Film

**Figure 5:**
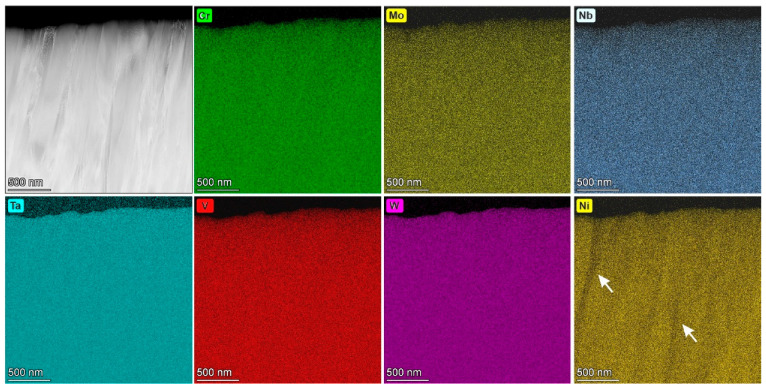
HAADF STEM image of the HEA thin film with elemental EDX maps. Arrows mark Ni-poor areas.

An HAADF STEM image of the HEA thin film and elemental EDX maps are presented in Figure 5. EDX measurements of the HEA thin film in TEM on the small scale show almost equal atomic concentrations of Cr, Mo, Nb, Ta, V and W elements of around 15–17 at. % with uniform distribution, akin to that we observed in SEM on the large scale (Table 1). On the Ni map, there are regions with changing Ni concentrations, which are indicated by arrows in Figure 5. The local measurements of Ni concentration in the thin film are in the range of 0.5 at. % to 4.7 at. %. Considering the average thickness in TEM samples along the electron beam, regions without Ni could exist.

Note that the constituting metals Cr, Mo, Nb, Ta, V and W originally have BCC crystal structures, while Ni has face-centered cubic crystal structure. From a thermodynamic equilibrium point of view, Ni has different solubilities with the individual metals and it may not match with the BCC crystal structure of the investigated HEA thin film. Moreover, the solubility of Ni may be low for such complex systems as the 6-elemental HEA. Considering the kinetics of thin film formation in physical vapor deposition, especially in magnetron sputtering processes (i.e., reflecting very high cooling rates up to 10^13^ K/s from the vapor phase), the solubility of Ni in such alloys could be enhanced. The key question is up to what concentration Ni can be dissolved in the BCC structure and whether it is energetically more favorable for Ni to be dissolved in the BCC crystal or to accumulate at the grain boundaries. These three factors may contribute to the inhomogeneity of the Ni-distribution, which we observe in elemental EDX maps in Figure 5.

**Figure 6:**
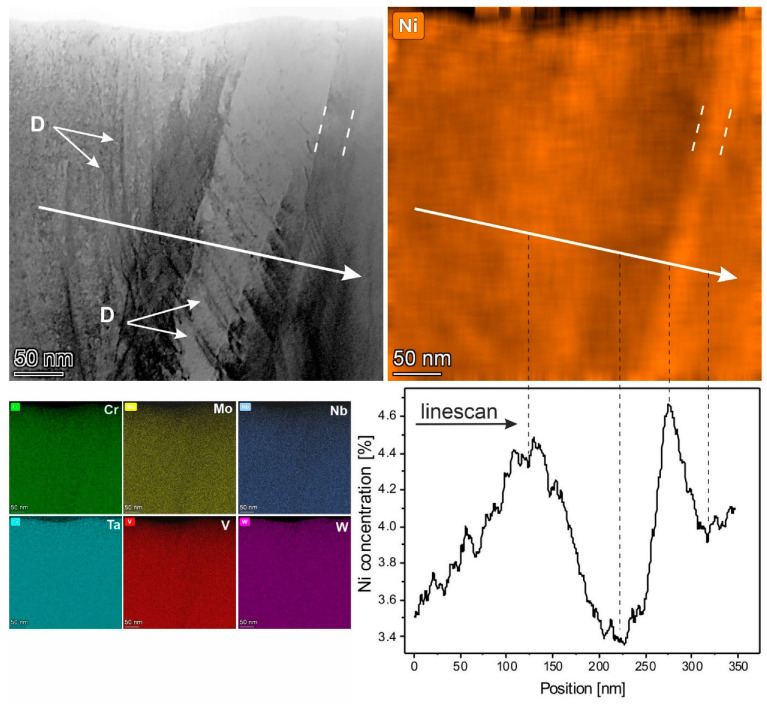
Inverted HAADF STEM image of the HEA thin film with elemental EDX maps. The arrows mark a linescan of the measured Ni-concentration. The boundaries of Ni-rich areas are indicated by white dashed lines. The maximums and minimum of the Ni-concentration in the linescan are marked by black dashed lines. White arrows are annotated as D mark dislocations.

For the determination of an exact location of Ni, inside grains or at grain boundaries, an EDX analysis on larger scale was carried out. This is presented in Figure 6.

As is the case in Figure 3, in the inverted HAADF STEM image in Figure 6, there are groups of dislocations, which are reflected by dark lines and marked by white arrows and annotated as D. In the EDX maps, beside the almost homogeneous distribution of the elements Cr, Mo, Nb, Ta, V and W, we observe a changing Ni concentration in the range of 3.35 at. % to 4.65 at. % perpendicular to the columnar grains. Two peaks of maximums of the Ni concentration from the right-hand side are observed in ~50 nm wide regions, whereas regions with increasing Ni-concentration from left hand and decreasing Ni-concentration in the middle of the linescan are more than 200 nm wide. The existence of Ni-rich areas with a small variation of only ca. 1% with sizes comparable with the grain width observed in Figure 6 does not allow us to reach a final conclusion about a possible Ni-segregation at the grain boundaries in the investigated HEA thin film. Note that the distributions of Cr, Mo, Nb, Ta, V and W along the linescan also vary in the range up to 2%. Moreover, it was shown in [11] that the solubility of Ni in similar conditions (but only in three-component NbMoTa refractory HEA) occurs up to 45 at. % over a wide range of temperatures. Therefore, and considering the present XRD and TEM analyses shown, we suggest that, in our investigated HEA thin film, grains with a different Ni concentration exist. The structural model for the HEA thin film in this investigation is a single-phase solid solution in the BCC structure with 3.6 at. % Ni incorporated in the BCC crystal.

### 3.4. Mechanical Properties

The Vickers hardness was 1390 HV and the reduced modulus of elasticity was 323 GPa. The dispersion of the measured values was very low. The standard deviation of 9 samples for the Vickers hardness was 43 HV and the standard deviation for the reduced modulus of elasticity was 14 GPa. This means that the Vickers hardness values are higher than those of hardened or nitrided steel and are comparable to the hardness of CrN coatings. In the four-component system MoNbTaW, Melia et al. [28] were able to achieve hardness values of up to 550 HV. This clearly demonstrates the advantage of the Ni-alloyed higher-component system in combination with a compressive residual stress. When examining the hardness impressions under a microscope, no cracking can be detected. No cracks can be found on the surface of the coating either. This shows that adding all elements during the system process is advantageous over subsequent alloying. However, it must be investigated whether cracks form during thermal post-treatment at higher temperatures. Zhang et al. [11] observed this. Four Si substrates were coated, and the residual stress and standard deviation were determined. The compressive residual stress was 777 GPa and the standard deviation was 140 GPa. These compressive properties cause an increase in Vickers hardness.

## 4. Conclusions

In this study, a novel, complex refractory Ni-containing CrMoNbTaVW HEA thin film was investigated. We focused on investigating the microstructure. In the six-component material system CrMoNbTaVW, all individual elements form a BCC structure. Ni itself forms FCC crystals. Nevertheless, at a concentration of about 3.6 at. % Ni, Ni could be completely dissolved in the BCC structure, thereby further increasing the entropy. This is a promising approach for designing a material, which could be applied in a more general way for novel HEAs. The thin film discussed is a single-phase film with a BCC structure, although the individual elements can vary from grain to grain by up to 2 at. %. With the low average Ni concentration of 3.6 %, the absolute fluctuation is most noticeable. No increase or decrease in Ni concentration directly at the grain boundaries could be detected. Furthermore, no diffraction signals of an FCC structure were found, even with a longer measurement time. The formation of Ni nanoclusters could not be detected within the resolution range. However, this will be investigated in the future using atomic probe measurements. The HEA thin film has columnar grains mainly in the {110}, {211} and {321} directions. The diameter of the columns varies between 40 nm and 400 nm. The defects observed in the HEA thin film are mainly single or an assembly of dislocations; they are mainly caused by stresses of the thin films resulting from film growth. This article presents the first major result of a significantly wider research study that will investigate the influence of Ni concentrations on the formation of the microstructure of Ni-containing refractory alloys over a wider range. Furthermore, the properties and behavior of such materials will be analyzed, especially the thermal and thermochemical stability of the Ni-containing single-phase BCC solid solution materials.

The next step will involve a rabid annealing series (200–900 °C, 1 h) followed by XRD/STEM, which will be used to quantify the metastability window; nano-indentation strain-rate jump tests or substrate–curvature measurements during thermal cycling should be performed to verify whether the stress is sufficient to activate the observed dislocation arrays and whether it changes the hardness or crack-initiation resistance. Atom-probe tomography (APT) will be carried out to decide whether these fluctuations are random solid-solution noise or embryonic Ni-rich clusters; the latter would act as heterogeneous nucleation sites during subsequent grain growth or phase separation. APT would also clarify whether Ni is truly intra-granular or segregated at dislocation cores.

## Figures and Tables

**Table 1:** SEM-EDX analyses: atomic concentrations of all elements in areas 1 and 2 of the HEA thin film from Figure 1a.

Element	Cr	Mo	Nb	Ta	V	W	Ni
at. %, 1	15.90	15.20	15.36	17.06	16.57	16.36	3.56
at. %, 2	15.74	15.11	15.42	17.34	15.44	17.30	3.65

## Data Availability

The original contributions presented in this study are included in the article. Further inquiries can be directed to the corresponding author.
